# Modified Pilates as an adjunct to standard physiotherapy care for urinary incontinence: a mixed methods pilot for a randomised controlled trial

**DOI:** 10.1186/s12905-017-0503-y

**Published:** 2018-01-12

**Authors:** Adi Lausen, Louise Marsland, Samantha Head, Joanna Jackson, Berthold Lausen

**Affiliations:** 1 Department of Affective Neuroscience and Psychophysiology, Institute for Psychology, University of Goettingen, Gosslerstr. 14, 37073 Goettingen, Germany; 2 Department of Mathematical Sciences, University of Essex, Wivenhoe Park, Colchester, CO4 3SQ UK; 3 School of Health & Social Care, University of Essex, Wivenhoe Park, Colchester, CO4 3SQ UK; 4 Physiotherapy Department, Anglian Community Enterprise (ACE) Community Interest Company, 910 The Crescent, Colchester Business Park, Colchester, CO4 9YQ UK; 5 School of Sport, Rehabilitation and Exercise Sciences, University of Essex, Wivenhoe Park, Colchester, CO4 3SQ UK

**Keywords:** Urinary incontinence, Modified Pilates, Physiotherapy, Pelvic floor muscle training, Mixed methods, Randomised controlled trial

## Abstract

**Background:**

Urinary incontinence (UI) is a distressing condition affecting at least 5 million women in England and Wales. Traditionally, physiotherapy for UI comprises pelvic floor muscle training, but although evidence suggests this can be effective it is also recognised that benefits are often compromised by patient motivation and commitment. In addition, there is increasing recognition that physical symptoms alone are poor indicators of the impact of incontinence on individuals’ lives. Consequently, more holistic approaches to the treatment of UI, such as Modified Pilates (MP) have been recommended. This study aimed to provide preliminary findings about the effectiveness of a 6-week course of MP classes as an adjunct to standard physiotherapy care for UI, and to test the feasibility of a randomised controlled trial (RCT) design.

**Methods:**

The study design was a single centre pilot RCT, plus qualitative interviews. 73 women referred to Women’s Health Physiotherapy Services for UI at Colchester Hospital University NHS Foundation Trust were randomly assigned to two groups: a 6-week course of MP classes in addition to standard physiotherapy care (intervention) or standard physiotherapy care only (control). Main outcome measures were self-reported UI, quality of life and self-esteem at baseline (T1), completion of treatment (T2), and 5 months after randomisation (T3). Qualitative interviews were conducted with a subgroup at T2 and T3. Due to the nature of the intervention blinding of participants, physiotherapists and researchers was not feasible.

**Results:**

Post-intervention data revealed a range of benefits for women who attended MP classes and who had lower symptom severity at baseline: improved self-esteem (*p* = 0.032), decreased social embarrassment (*p* = 0.026) and lower impact on normal daily activities (*p* = 0.025). In contrast, women with higher symptom severity showed improvement in their personal relationships (*p* = 0.017). Qualitative analysis supported these findings and also indicated that MP classes could positively influence attitudes to exercise, diet and wellbeing.

**Conclusions:**

A definitive RCT is feasible but will require a large sample size to inform clinical practice.

**Trial registration:**

ISRCTN74075972 Registered 12/12/12 (Retrospectively registered).

**Electronic supplementary material:**

The online version of this article (10.1186/s12905-017-0503-y) contains supplementary material, which is available to authorized users.

## Background

Urinary Incontinence (UI) is a distressing, socially embarrassing condition affecting at least 5 million women in England and Wales [1]. Feelings of low self-esteem, embarrassment and helplessness are commonly reported [2], together with withdrawal from social situations [3, 4]. In addition, UI is an important barrier to regular physical and fitness activities [5–7] and this withdrawal may threaten women’s general health and wellbeing [8]. It is estimated that ill-health attributable to physical inactivity costs the National Health Service (NHS) more than £1.06 billion per year and accounts for 16.9% of premature mortality in the UK [9]. The socioeconomic costs of UI are likely to extend beyond the immediate symptoms and may escalate as life expectancy increases [10].

Physiotherapy is important first line management for UI; many women wish to avoid invasive surgical procedures if possible, plus physiotherapy may provide a cheaper treatment solution for the NHS. The traditional form of physiotherapy for UI is pelvic floor muscle training (PFMT) [1, 11–14]. Although a variety of randomised controlled trials have documented the positive effects of intensive PMFT [11], it has also been suggested that patient motivation and commitment play an important role in ensuring its effectiveness [15]. Also, there is increasing recognition that symptoms alone are poor indicators of the effect of incontinence on individuals’ lives [16]. Therefore, more holistic approaches to the treatment of UI have been recommended [16, 17].

As an addition to standard UI treatment, more recently Modified Pilates (MP) – a mind-body approach involving slow, controlled movements focusing on posture and breathing – appears to have been increasingly integrated into physiotherapy rehabilitation programmes [18]. Pilates is a form of exercise, involving a range of movements that both strengthen and increase flexibility of the whole body, rather than having a specific muscle focus. MP avoids intense abdominal contractions, breath holding or straining that could put increased pressure on the pelvic floor while at the same time incorporating exercises that can incidentally train the pelvic floor [19]. However, the effectiveness of this approach is purely anecdotal with a lack of empirical evidence [20]. A recent systematic review of RCTs identified only two other studies, which aimed to assess the effectiveness of Pilates training in women suffering from UI [19]. Both these studies, however, focused on Pilates provided individually (as opposed to in class sessions) and neither provided sufficient data to permit inferences about the effects of MP [11, 13].

It was apparent therefore that a trial to evaluate the effectiveness of a course of MP classes as an adjunct to standard physiotherapy care for UI with an initial pilot study was required. Specifically, this pilot study aimed to: (1) assess the feasibility of the trial protocol to evaluate the effectiveness of a course of MP classes as an adjunct to standard physiotherapy care for UI; (2) assess the variation of the main outcome measures in order to inform sample size considerations for a full randomised controlled trial; (3) provide some preliminary data about the effectiveness of MP classes; (4) identify the benefits/limitations and acceptability of standard physiotherapy care plus MP classes compared to standard physiotherapy care alone. To the best of our knowledge, this research is the first to fully evaluate and report on the effectiveness of MP classes as part of the physiotherapy management of women with UI.

## Method

### Participants

**Fig. 1:**
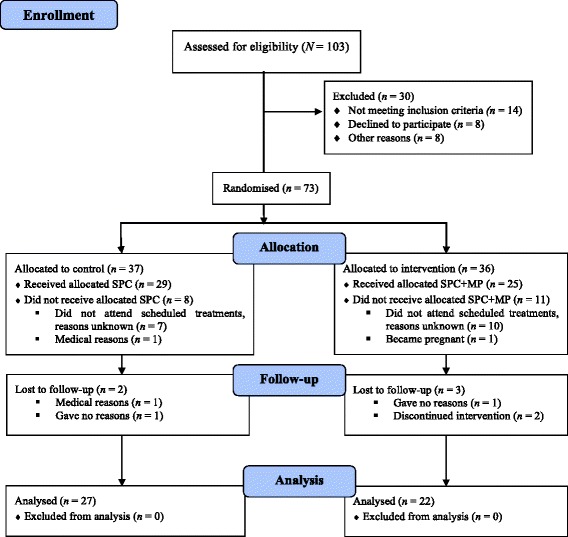
Flowchart of study participants (Study period: November 2012–October 2014)

103 women referred for physiotherapy for UI by their General Practitioner, consultant or continence nurse were assessed for eligibility to participate in the trial. The inclusion criteria for taking part in the study were to be aged above 18 and diagnosed with stress, urge or mixed UI. Participants were not eligible to participate if they had a history of pelvic malignancy, were suffering from faecal incontinence, central nervous system diseases, had given birth in the previous 3 months or undergone gynaecological surgery in the previous 6 months. 30 women were excluded or withdrew before randomisation (14 did not meet the inclusion criteria, eight declined to participate and eight withdrew citing lack of time or other reasons). A total of 73 women (*M*_*age*_ = 50.11, *SD* = 13.18), were randomly allocated to an intervention or a control group. The control group (*n* = 37, *M*_*age*_ = 48.97, *SD* = 12.25) received standard physiotherapy care (SPC) only (i.e., pelvic floor exercises and lifestyle advice), whereas the intervention group (*n* = 36, *M*_*age*_ = 51.28, *SD* = 14.15) attended a 6-week course of MP classes in addition to SPC (SPC + MP). Fig. 1 shows the study’s flow diagram according to CONSORT guidelines (Fig. 1 Flowchart of study participants).

### Outcome measures

As this was a pilot study it was appropriate to explore a range of outcome measures (aims 1, 2, 3). Accordingly, several standardised self-report measures were used to assess participants’ symptoms of incontinence (Symptom Severity Index (SSI) [21]), their impact on quality of life (Incontinence Quality of Life Questionnaire (I-QOL) [22]; ICIQ – Lower Urinary Tract Symptoms Quality of Life (ICIQ-LUTSqol) [23, 24]), and self-esteem (Rosenberg Self-Esteem Scale (RSE) [25]). These measures were used in preference to clinical measures such as pad tests or pelvic muscle strength because the focus of this study was the psychological and social impact of UI on women’s lives rather than level of incontinence alone. Furthermore, the project Public and Patient Involvement (PPI) group advised against the burden of including such clinical measures. The PPI group comprised women who had been previously treated for UI and who provided advice throughout the project to ensure the research was informed by their views and experiences. Both the intervention and control groups completed the questionnaires at baseline (T1), the end of treatment (T2) and 5 months after randomisation (T3). For a brief scoring description of the questionnaires see *Outcome Measures* (in Additional file 1).

In addition, qualitative interviews were conducted with a subgroup of 16 participants (8 SPC and 8 SPC + MP) at T2 and T3 to explore unanticipated benefits/limitations and acceptability of SPC + MP compared with SPC (aim 4). The interviews were undertaken by a qualitative researcher and were semi-structured in nature using a topic guide to ensure that all topics were covered with every participant but allowing varying levels of detail to emerge according to their particular responses. Interviews at T2 lasted between 45 min and 1 h, while those at T3 tended to be shorter, averaging around 30 min.

### Design

A single centre, pilot randomised controlled trial (RCT) carried out over 24 months, plus qualitative interviews with a subgroup of participants.

### Randomisation

Prior to randomisation, stratification by body mass (BMI) and symptom severity indices (SSI) was undertaken since BMI and SSI are known predictors for the successful treatment of UI in women [26, 27]. Randomisation was by computer allocation using the web-based randomisation service of the Norwich Clinical Trials Unit. To ensure the treatment groups were similar a stratified randomisation with 3 strata (i.e., low SSI; high SSI/low BMI; high SSI/high BMI) was used. In each stratum, balanced blocks of random length (4, 6 or 8) were fitted to assign an equal number of SPC + MP and SPC participants. Due to the nature of the intervention, blinding of participants, physiotherapists and researchers was not feasible.

### Setting

The research was conducted at the Women’s Health Physiotherapy Service, Colchester Hospital University NHS Foundation Trust (CHUFT).

### Procedure

Participants were invited to take part in the study in which they would undertake either SPC or SPC + MP offered by the Women’s Health Physiotherapy Service at CHUFT. The study was approved by the National Research Ethics Service (NRES) East of England Hertfordshire Ethics Committee.

After being screened by the Chief Investigator or project administrator (PA) for eligibility to take part in the study, the PA telephoned the women to arrange their first clinical appointment. During this call the PA explained that, together with confirmation of their appointment, they would also be sent details about the research and the *Participant Information Sheet*. A telephone number, email address and reply slip with pre-paid envelope was provided for those seeking additional information about the research and/or to indicate interest in participation. Those women who agreed to take part provided written informed consent during their first clinical appointment (SPC session 1). Consented participants then met with the research physiotherapist for assessment of their ability to contract pelvic floor muscles (through vaginal digital palpation) and to establish whether they had sufficient mental capacity to complete the questionnaires and/or follow exercise instructions (according to the principles of the Mental Capacity Act 2005 [28]). T1 data were collected by the research physiotherapist at the SPC session 2. T2 and T3 data were collected via questionnaire packs sent by post enclosing a stamped addressed envelope for their return.

**Fig. 2:**
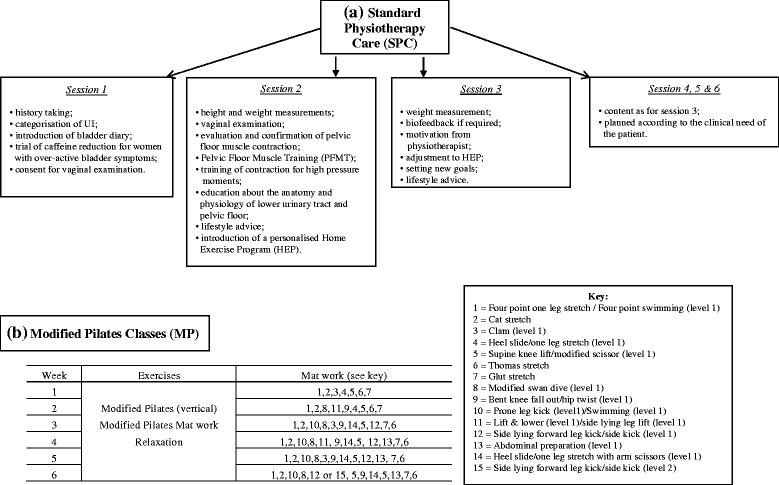
**a** Overview of the Standard Physiotherapy Care (SPC) sessions; (**b**) Overview of the Modified Pilates (MP) classes

The SPC treatment for UI provided by CHUFT Women’s Health Physiotherapy Service comprised 3 to 6 individual sessions over a 3–6 months’ period. The NICE guidelines for UI recommend supervised pelvic floor muscle training of at least 3 months’ duration. If pelvic floor muscle contraction has been confirmed, then women are normally offered this supervision via three appointments over the time period [1]. SPC sessions included PFMT, biofeedback, a home exercise programme, and lifestyle advice. Participants allocated to the SPC + MP intervention received 3 SPC sessions ahead of the MP classes, with further (up to a total of 6) SPC sessions arranged during or subsequent to the course of MP classes dependent upon clinical need. The MP intervention consisted of 6 one-hour group classes, of 6–8 women, run at one-week intervals by a physiotherapist with specialist qualifications in Pilates. The focus of the MP exercises was on low intensity abdominal and pelvic floor control and on the awareness of posture and breathing thought to promote a mind-body connection. The instructor guided the class through a continuous flow of low level exercises while keeping patients engaged with their mind-body connection by using descriptive cueing and visualisation techniques (for details regarding content of SPC and SPC + MP see Fig. 2 (a) Overview of the Standard Physiotherapy Care (SPC) sessions; (b) Overview of the Modified Pilates (MP) classes).

### Qualitative and quantitative analyses

A lack of existing data about the effectiveness of MP delivered in a group setting meant that precise sample size estimates were not possible. A sample size of 100 was therefore determined since it was anticipated that 50 participants per arm would enable detection of a difference of 10 with a standard deviation (SD) of 15 on quality of life (QoL) measurements (*I-QOL* and *ICIQ-LUTSqol*), and a difference of 2 with a SD of 3 on the SSI with a power of 70% for a Bonferroni adjusted 5% significance level [29]. Assumptions regarding QoL and SSI indices were justified by Lamb et al. [30]; Rosenberg self-esteem index [25] was assumed to follow a similar pattern. Calculations allowed for 20% attrition, i.e. 40 participants in each group with completed measurements.

Statistical analyses were performed using the R language and environment for statistical computing and graphics v 3.3.1 [31]. As the data were ordinal and discrete and did not justify the assumption of normal distribution Wilcoxon-Mann-Whitney test was used to assess the baseline characteristics of the study population (aim 2), to test the differences of pre- and post-tests of all outcome measures between the two groups SPC + MP and SPC (aim 3) and to evaluate the impact of SPC + MP in comparison to SPC in the study population and in the strata low SSI and high SSI (aim 3). To quantify the size of the difference between the two groups SPC + MP and SPC Cohen’s standardised effect size was calculated in the strata (aim 3). Describing the reliability of the questionnaires was considered an important aspect of exploring the feasibility of the study protocol (aim 1) since even when outcome measures are standardised and well-published it cannot be assumed that they are reliable within a particular target population.

In addition, 16 women (8 from each group) were interviewed at T2, and 15 at T3 (1 withdrew). The sample was selected to reflect diversity across a number of variables: age, ethnicity, BMI and SSI (aim 4). All interviews were recorded, transcribed verbatim, and analysed using the principles of *Framework* [32, 33]. A coding frame of key codes and categories was created, and the transcripts coded within *MaxQda*, a software package specifically designed to assist with the analysis of qualitative data. The rigour of this process was enhanced by independent review of the transcripts and categorisation of main themes to emerge by the qualitative researcher, principal investigator and research physiotherapist [34].

## Results

Observations with missing data were included in the analysis based on the following criteria: for average scores, each observation had to have no more than one unanswered item, while simple sum scores were created using only those observations with complete responses. For all analyses, a two-tailed *p*-value less than 0.05 was considered statistically significant and a p-value between 0.05 and 0.1 was interpreted as indicating a trend.

### Baseline characteristics of the study population

There were no significant differences in terms of mean and standard deviation for age, BMI, SSI, self-esteem or QoL indicating that at baseline the SPC and SPC + MP groups were similar and randomisation worked well (see Table 1).

**Table 1:** Median (Md), interquartile range (IQR), mean (M), standard deviation (SD) and *p*-values of all pre-test measurements

Variables	Dimensions	Group										
		SPC					SPC + MP					
		*n*	*Md*	*IQR*	*M*	*SD*	*n*	*Md*	*IQR*	*M*	*SD*	*p-value*
Age		37	50.00	21.00	48.97	12.25	36	50.50	20.50	51.28	14.15	0.53
BMI		37	28.36	5.64	28.63	4.69	36	28.35	6.50	28.69	4.77	0.80
SSI		37	13.00	7.00	12.53	4.09	36	12.25	8.00	11.45	4.54	0.34
SII		37	6.00	3.00	6.60	3.11	36	5.00	3.00	5.67	2.37	0.24
RSE		37	20.00	10.00	20.58	6.26	36	23.00	5.25	22.44	5.15	0.16
*I-QOL*	ALB^b^	37	56.25	37.50	51.10	23.43	36	46.88	31.25	49.83	22.05	0.72
	PS^b^	37	66.67	44.44	57.28	27.83	36	65.28	42.36	62.27	22.99	0.43
	SE^b^	37	30.00	45.00	32.84	26.13	36	27.50	41.25	34.17	26.66	0.84
	Total^a^	37	52.27	39.77	49.48	23.27	36	49.43	33.81	51.36	21.21	0.71
*ICIQ-UI sf.*		37	13.00	8.00	12.68	4.66	34	12.50	7.00	12.18	4.75	0.59
*ICIQ-LUTSqol*	Role limitations	37	50.00	50.00	45.05	31.64	36	41.67	33.33	48.61	28.56	0.70
	Physical limitations	37	50.00	33.33	50.90	24.20	36	50.00	33.33	51.85	26.06	0.99
	Social limitations	37	22.22	55.56	30.93	31.00	36	19.44	47.22	27.16	26.55	0.71
	Personal relationships	28	33.33	83.33	38.69	36.87	32	16.67	50.00	30.21	36.28	0.31
	Emotions	37	33.33	44.44	44.14	32.23	36	33.33	47.22	36.73	27.71	0.36
	Sleep/energy	37	33.33	33.33	42.34	26.23	36	33.33	0.00	37.04	22.22	0.30
	Severity measures	37	58.33	33.33	54.73	22.44	36	50.00	37.50	50.69	26.98	0.43
	Overall score^c^	37	38.60	34.86	45.66	21.92	36	36.16	26.46	41.17	22.05	0.36

*Note:* All group comparisons were made using the *Wilcoxon-Mann-Whitney* test. For the *I-QOL* total^a^, higher scores indicate greater quality of life, whereas for its subscales^b^, higher scores indicate less *ALB*, *PS* and *SE*. For the *ICIQ-LUTSqol* overall score^c^, higher scores denote increased impact on quality of life

*BMI* Body Mass Index, *SSI* Symptom Severity Index, *SII* Symptom Impact Index, *I-QOL* Incontinence Quality of Life Questionnaire, *ALB* Avoidance and Limiting Behaviour, *PS* Psychosocial Impacts, *SE* Social Embarrassment, *ICIQ-UI* *sf*. Incontinence Quality of Life Questionnaire - Urinary incontinence short form, *ICIQ-LUTSqol* Incontinence Quality of Life Questionnaire – Lower Urinary Tract Symptoms Quality of Life)

The psychometric properties of the main outcome measures showed excellent internal consistency (Cronbach’s α ≥ 0.9) for *Rosenberg Self-Esteem Scale* (RSE) and *International-Quality of Life Questionnaire* (I-QoL) and good internal consistency (0.7 ≤ α < 0.9) for *Symptom Severity Index* (SSI) and *International Consultation on Incontinence Questionnaire – Lower Urinary Tract Symptoms Quality of Life* (ICIQ-LUTSqol).

### Outcome measures main analysis

From the 36 women allocated to the intervention (SPC + MP) group and the 37 women allocated to the control (SPC group), 14 and 10 women respectively were removed from the complete case analysis for the following reasons: failed to attend the scheduled treatments (10 SPC + MP; 7 SPC), medical reasons (1 SPC + MP; 1 SPC), lost to follow-up (3 SPC + MP; 2 SPC). This left a total of 49 participants with a mean age 52.14 (*SD* = 11.67). The SPC group (*n* = 27) had a mean age of 51.11 years (*SD* = 10.76) and the SPC + MP group (*n* = 22) a mean age of 53.41 years (*SD* = 12.85). Medians, interquartile range, means, standard deviations and *p*-values of all pre-test measurements with complete follow-up are shown in Table 2. No differences between the two groups, except a trend for symptom severity index (*p* = 0.08) which was lower in the intervention (SPC + MP) than in the control group (SPC), were observed.

**Table 2:** Median (Md), interquartile range (IQR), mean (M), standard deviation (SD) and p-values of all pre-test measurements with complete follow-up

Variables	Dimensions	Group										
		SPC					SPC + MP					
		*n*	*Md*	*IQR*	*M*	*SD*	*n*	*Md*	*IQR*	*M*	*SD*	*p-value*
Age		27	51.00	16.00	51.11	10.76	22	52.00	21.50	53.41	12.85	0.55
BMI		27	27.55	6.15	28.60	5.08	22	26.68	7.21	27.89	5.33	0.74
SSI		27	13.00	5.13	13.17	3.65	22	11.50	6.75	10.84	4.70	0.08
SII		27	6.00	3.50	6.74	3.10	22	5.00	2.75	5.55	2.42	0.16
RSE		27	20.00	10.00	20.76	6.50	22	24.00	5.00	23.27	4.34	0.18
*I-QOL*	ALB^b^	27	53.12	34.38	48.61	20.03	22	46.88	28.13	49.72	21.37	0.98
	PS^b^	27	66.67	45.83	56.79	26.23	22	69.44	43.75	62.25	23.98	0.37
	SE^b^	27	30.00	37.50	31.85	24.97	22	30.00	38.75	34.55	23.09	0.66
	Total^a^	27	51.14	39.77	48.15	21.24	22	51.14	32.67	51.39	20.58	0.58
*ICIQ-UI sf.*		27	13.00	6.50	12.56	4.68	22	12.50	8.00	11.68	5.29	0.51
*ICIQ-LUTSqol*	Role limitations	27	50.00	41.67	44.44	31.35	22	33.33	33.33	49.24	30.64	0.71
	Physical limitations	27	50.00	33.33	52.47	22.98	22	41.67	29.17	48.48	27.17	0.40
	Social limitations	27	22.22	38.89	27.57	29.62	22	22.22	47.22	28.79	27.30	0.79
	Personal relationships	20	33.33	70.83	40.83	36.86	19	16.67	83.33	37.72	41.52	0.62
	Emotions	27	33.33	33.33	41.56	29.17	22	27.78	44.44	35.86	28.26	0.48
	Sleep/energy	27	33.33	33.33	45.68	24.28	22	33.33	12.50	39.39	24.48	0.28
	Severity measures	27	58.33	16.67	56.17	17.69	22	45.83	39.58	50.76	27.81	0.36
	Overall score^c^	27	38.60	26.64	45.55	18.63	22	31.58	28.59	41.67	23.64	0.29

*Note:* All group comparisons were made using the *Wilcoxon-Mann-Whitney* test. For the *I-QOL* total^a^, higher scores indicate greater quality of life, whereas for its subscales^b^, higher scores indicate less *ALB*, *PS* and *SE*. For the *ICIQ-LUTSqol* overall score^c^, higher scores denote increased impact on quality of life

*BMI* Body Mass Index, *SSI* Symptom Severity Index, *SII* Symptom Impact Index, *I-QOL* Incontinence Quality of Life Questionnaire, *ALB* Avoidance and Limiting Behaviour, *PS* Psychosocial Impacts, *SE* Social Embarrassment, *ICIQ-UI sf*. Incontinence Quality of Life Questionnaire - Urinary incontinence short form, *ICIQ-LUTSqol* Incontinence Quality of Life Questionnaire – Lower Urinary Tract Symptoms Quality of Life

The control group (SPC) had an average of 5.63 SPC sessions (*Md* = 6 sessions), whereas the intervention group (SPC + MP) had an average of 4.27 SPC sessions (*Md* = 4 sessions). The length of time between SPC session 1 and session 2 was *M* = 12.92 days (*Md* = 11 days). The average time between the first and the last SPC session was 167.60 days (*Md* = 177 days) for the SPC group and 135.00 days (*Md* = 152 days) for the SPC + MP group.

The pre- post-test measurements of the main outcome variables (primary analyses) showed no significant differences between the two groups (Table 3).

**Table 3:** Median (Md), interquartile range (IQR), mean (M), standard deviation (SD) and p-values of all pre- post-test differences

Variables	Dimensions	Group										
		SPC					SPC + MP					
		*n*	*Md*	*IQR*	*M*	*SD*	*n*	*Md*	*IQR*	*M*	*SD*	*p-value*
SSI		27	−3.00	4.50	−2.32	3.76	22	−1.50	3.38	−1.99	3.00	0.58
SII		27	−2.00	3.50	−1.82	2.06	22	0.00	3.00	−0.82	1.94	0.08
RSE		27	0.00	4.50	−0.20	2.99	22	1.00	4.50	0.86	3.32	0.21
*I-QOL*	ALB^b^	27	9.38	20.31	14.70	17.97	21	12.50	21.88	16.82	20.76	0.47
	PS^b^	27	8.33	16.67	8.58	16.40	22	13.88	19.53	15.55	22.69	0.15
	SE^b^	27	15.00	25.00	15.00	19.36	22	22.50	27.50	23.86	24.39	0.10
	Total^a^	27	12.50	21.59	12.23	15.89	21	15.91	18.24	17.96	21.10	0.18
*ICIQ-UI sf.*		22	−3.00	3.75	−3.27	2.57	17	−3.00	5.00	−3.06	4.13	0.51
*ICIQ-LUTSqol*	Role limitations	27	−16.67	25.00	−15.43	23.99	22	−16.67	16.67	−21.97	22.65	0.098
	Physical limitations	27	0.00	33.33	−12.96	25.04	22	−16.67	33.33	−14.39	29.23	0.83
	Social limitations	27	0.00	16.67	−9.47	28.86	22	−13.89	33.33	−18.69	25.11	0.13
	Personal relationships	19	0.00	8.33	−8.77	26.86	19	−16.67	25.00	−14.91	39.24	0.16
	Emotions	27	−11.11	22.22	−8.64	23.33	22	−11.11	27.78	−10.61	24.36	0.91
	Sleep/energy	27	−16.67	33.33	−9.88	23.23	22	−8.33	16.67	−6.06	19.62	0.74
	Severity measures	27	−8.33	16.67	−6.48	16.23	22	−8.33	16.67	−10.61	16.50	0.28
	Overall score^c^	27	−9.26	11.35	−10.76	16.85	22	−10.87	17.25	−13.38	17.80	0.44

*Note:* All group comparisons were made using the *Wilcoxon-Mann-Whitney* test. For the *I-QOL* total^a^, higher scores indicate greater quality of life, whereas for its subscales^b^, higher scores indicate less *ALB*, *PS* and *SE*. For the *ICIQ-LUTSqol* overall score^c^, higher scores denote increased impact on quality of life

*BMI* Body Mass Index, *SSI* Symptom Severity Index, *SII* Symptom Impact Index, *I-QOL* Incontinence Quality of Life Questionnaire, *ALB* Avoidance and Limiting Behaviour, *PS* Psychosocial Impacts, *SE* Social Embarrassment; *ICIQ-UI sf*. Incontinence Quality of Life Questionnaire - Urinary incontinence short form, *ICIQ-LUTSqol* Incontinence Quality of Life Questionnaire – Lower Urinary Tract Symptoms Quality of Life

### Outcome measures main analysis in strata

**Fig. 3:**
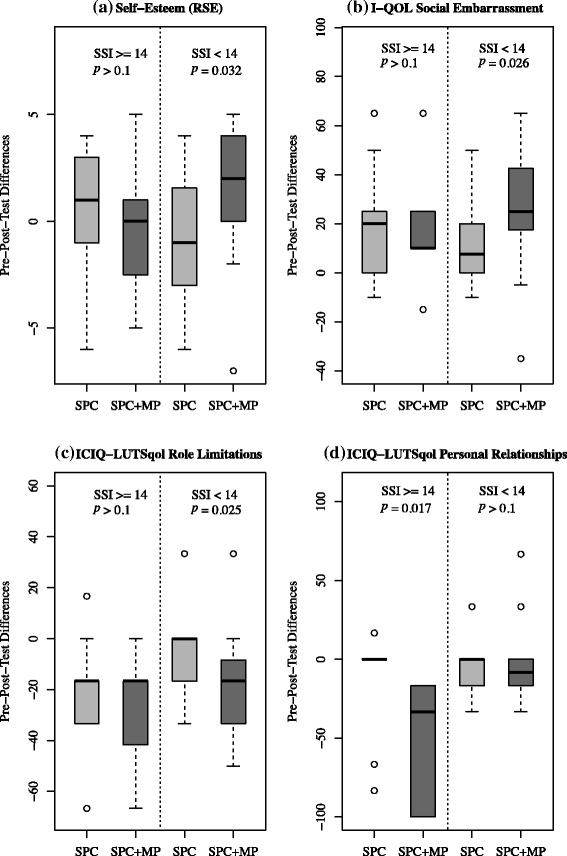
Boxplots of pre- and post-tests differences of (**a**) self-esteem, (**b**) social embarrassment (**c**) normal daily activities and (**d**) social life between pre- and post-tests for low and high SSI. Note: On the y-axis, positive values of pre- post-test differences indicate improved self-esteem [Rosenberg Self-Esteem (RSE)] and reduced embarrassment [Incontinence quality of life questionnaire (I-QOL) – Social Embarrassment (SE)], whereas the negative values indicate that the women were less affected by the incontinence in their normal activities, e.g., job [International Consultation on Incontinence Questionnaire - Lower Urinary Tract Symptoms Quality of Life (ICIQ-LUTSqol) - Role limitations]

Post-intervention participants in the SPC + MP group with low SSI showed significant increases in self-esteem (standardized effect size *d* = 0.79, *p* = 0.032), felt less social embarrassment (*d* = 0.73, *p* = 0.026) and were less affected by UI in their normal daily activities (*d* = −0.73, *p* = 0.025) when compared to the SPC group. Furthermore, positive trends in SPC + MP group indicated that, post-intervention, women with low SSI had a higher QoL overall score (*d* = 0.65, *p* = 0.052) and their social life was less impacted by the UI symptoms (*d* = −0.60, *p* = 0.089) in comparison to the women in the SPC group. In contrast, SPC + MP group participants with high SSI showed significant improvements in their personal relationships (*d* = −1.10, *p* = 0.017) between pre- and post-tests when compared with those receiving SPC only. The boxplots for the differences between pre- and post-tests of self-esteem and QoL dimensions illustrate the combined effect of SSI and MP between T1 and T3 (See Fig. 3: Boxplots of pre- and post-tests differences of (a) self-esteem, (b) social embarrassment (c) normal daily activities and (d) social life for both groups (SPC and SPC + MP) in the low and high SSI strata).

### Qualitative findings

From the information provided by women attending SPC only, it appeared that these sessions were individually tailored but always included detailed PFMT. Whilst benefits were reported in terms of some improvement in physical symptoms, by T3 a plateau appeared to have been reached, and even those most committed to continuing PFMT described feeling ‘demoralised’ or doubting the effectiveness of this approach to treatment long term.

Allocation to the SPC + MP group was generally met with enthusiasm, and women in this group seemed aware of the general health benefits of Pilates. Overall, experiences of the classes were positive and related to: the gentle approach to exercise; undertaking exercises in different positions; frequency and intensity of undertaking the exercises, and a sense of fun and enjoyment. A number of aspects appeared to impact positively on the women’s emotional experience, in particular the group support encountered, and a sense of ‘feeling safe’ both physically and emotionally. Improvements in symptoms reported by the SPC + MP group resulted both from a perceived increase in the strength of pelvic floor muscles and a greater awareness of how and when to use pelvic floor muscles to avoid leakage in everyday situations. Some concern and disappointment was also expressed that improvements were not as great as had been hoped, or required conscious management, and an association emerged from the data between SSI and the perceived effectiveness of MP classes. In addition to changes in UI symptoms, some women, in particular those with higher self-esteem, spoke about the value of the MP classes in terms of what it taught them about themselves and their bodies, for example motivating them to continue looking after themselves through increased exercise and attention to diet. Others also described increased confidence in undertaking activities such as going out for a walk. In terms of the acceptability of MP classes, a number of suggestions were made. These included: the importance of using everyday language rather than medical terms; a preference to know whether all group members suffered from UI; the importance of continuity between SPC and treatment provided in the MP classes; the potential value of ‘take home’ instructions, and some practical issues such as the timing of groups and facilities available.

## Discussion

The quantitative results of this pilot trial confirmed that the baseline characteristics of the two groups were similar (i.e., that the randomisation worked) and the outcome measures had high reliability (Cronbach’s α > 0.80). Data revealed a range of benefits for women who attended MP classes in addition to SPC (improved self-esteem, decreased social embarrassment, less impact on normal daily activities and improved personal relationships) especially for women with lower symptom severity. In addition, the qualitative analysis supported these findings and also indicated that MP classes could positively influence attitudes to exercise, diet and wellbeing. It is possible that some of the outcomes observed could be due to benefits of attending a class or group other than the MP exercises. However, the purpose of this pilot study was to evaluate the effectiveness of MP classes as a whole experience rather than attempt to isolate the effects of MP exercises, since it is the delivery of MP in a group or class setting that is most likely to prove cost effective for the NHS.

### Comparison with existing literature

Similar to previous studies [11, 13], our sample was not sufficiently powered to make clear inferences about the effectiveness of the intervention and, therefore, until further results are produced this approach should not be recommended for routine use in clinical practice. Unlike previous research, our pilot trial aimed to assess the feasibility of the study protocol (testing phase) in order to conduct a future high-quality RCT. Although the impact of including MP as an adjunct to SPC has not yet quite been established, the results of this study identify patterns that warrant further investigation. Therefore, with a number of modifications it appears feasible to transfer the research protocol of this pilot study to a larger multicentre trial.

### Strengths & limitations

This is the first clinical trial to assess the efficacy of MP classes in the management of UI using a mixed-method approach. The study provides new insights into the possible positive effects of the intervention, and to its implementation in practice. However, the study sample was not sufficiently powered to establish conclusively whether the addition of MP classes as an adjunct to SPC for women with UI is of benefit (i.e., this might have been only the result of chance accounted for in the statistical analysis). Moreover, the duration of the intervention was short relative to the duration of UI, making it difficult to reliably capture the efficacy of MP classes, and although the supervised SPC sessions were provided in accordance with NICE guidelines [1, 3], this was less frequent than in the research from which these guidelines were developed. Despite several modifications made to improve recruitment to the study (based on the feedback from potential participants and the project PPI group) another challenge posed by this trial was the retention rate at follow-up. Consequently a complete dataset was obtained for about two-thirds of the randomised patients.

The observation that 14 women of the SPC + MP group compared to 10 of the SPC only group withdrew from the study could challenge the assumption that MP classes have the potential to improve motivation and commitment to exercise believed to be beneficial for UI. Findings from the interviews and discussion with the project PPI group suggest however, that attrition from the MP + SPC group was most likely to be due to aspects of delivery, for example the timing of the classes and geographical location, rather than the nature of the exercises. Moreover, withdrawal from the study does not necessarily equate to cessation of exercise. It is therefore essential that aspects of delivery and subsequent adherence to exercise are fully explored in a definitive trial so that potential delivery in the NHS is fully informed.

This study utilised self-reported measures of symptom severity in preference to clinical measures such as pad tests or pelvic floor muscle strength. Whilst this could be viewed as a limitation, members of project PPI group were adamant that inclusion of such invasive measures could seriously impact on recruitment from this particular population of women attending Women’s Physiotherapy for UI. In addition, it should be noted that the NICE guidelines [1] do not recommend the use of pad tests in routine clinical assessment. Most importantly, however, members of the group emphasised that it was the psychological and social impact of UI on their lives and the potential benefits of treatments in this respect that required evaluating, rather than the level of incontinence per se. This study therefore makes an essential contribution to understanding the potential positive benefits of MP on the lives of women who experience UI. It is however recognised that exclusion of a clinical measure of leakage limits the impact of this research. Moving forward, intensive work with the PPI group will explore further the risk to recruitment of including such measures (given that recruitment proved to be problematic even without clinical measures), together with the relative importance to women experiencing UI of the degree of incontinence versus their overall sense of well-being. This work will inform the design of future research.

A further aspect to be considered in future designs is the inequality of the two interventions in terms of contact time. To fully understand the potential benefit of MP classes as a treatment for UI in terms of cost to the NHS, the amount and type of contact received by participants in the two groups will need to be equivalent.

### Future research

Given the significant results and positive trends indicated by this study, one important issue for future research is to assess the long-term effects of including MP classes within the management of UI. A future full RCT is therefore required, incorporating both a larger sample size and a more robust follow-up strategy (e.g., administering questionnaires face-to-face). Findings stemming from this pilot indicate that women with low SSI were most likely to experience benefits from adjunct MP sessions, however, the study does not provide the complete picture of what can be expected in clinical practice. Thus, future research could explore whether focusing only on women with a lower SSI makes greatest clinical and socioeconomic sense. Additional contributions could be made by comparing the effects of MP classes with other forms of therapy (e.g., cognitive-behavioural therapy) or other forms of group support, and also through considering the potential impact of MP classes on the number of SPC sessions that are required.

### Implications for practice

The use of both qualitative and quantitative methods to evaluate the effectiveness of MP delivered in a group setting, which until now has been based purely on anecdote, provides a greater understanding about such an approach and ensures that the voice of patients contributes to the development of practice. Given the negative impact of UI on social health and wellbeing, this type of exploration is greatly needed to fully understand the longitudinal impact of UI and reduce the psychological burden on patients and their family. Also, such an approach could provide an important alternative treatment for women wishing to avoid surgery, be cost effective, reduce impact on job participation and provide long lasting health benefits. Finally, it might serve as a point of departure for clinicians designing intervention strategies, which could help individuals affected by this condition develop the necessary skills and empowerment to help manage the challenges associated with incontinence.

## Conclusion

In summary, the global scores of the pre-post-test differences were comparable between the groups (SPC and SPC + MP). Secondary sub-groups analyses indicated that SPC + MP in comparison to SPC improved self-esteem, normal daily activities and reduced the feelings of embarrassment in women with low SSI, and for women with high SSI improved their personal relationships.

Taken altogether, the results of the pilot trial provide important parameter estimates to plan the recruitment, the sample size and the number of trial centres of the full trial. Moreover, the evaluation of the instruments allows the selection of an efficient set of questionnaires to assess the effectiveness of including Modified Pilates as an adjunct to standard physiotherapy care for women with urinary incontinence.

## Additional file

Additional file 1: Outcome measures - A description of the four outcome measures/questionnaires used: Symptom severity index (SSI)/Symptom Impact Index (SII); Incontinence quality of life questionnaire (I-QOL); International Consultation on Incontinence Questionnaire (ICIQ); Rosenberg self-esteem scale (RSE). (DOCX 25 kb)

## Abbreviations

*BMI*: Body mass index
*CHUFT*: Colchester Hospital University NHS Foundation Trust
*ICIQ-LUTSqol*: International Consultation on Incontinence Modular Questionnaire - Lower Urinary Tract Symptoms Quality of Life Questionnaire
*I-QOL*: Incontinence Quality of Life Questionnaire
*MP*: Modified Pilates
*NHS*: National Health Service
*NRES*: National Research Ethics Service
*PA*: Project administrator
*PFMT*: Pelvic floor muscle training
PPI: Public and patient involvement
*QoL*: Quality of life
*RCT*: Randomised controlled trial
*RSE*: Rosenberg Self-Esteem Scale
*SPC + MP*: Standard physiotherapy care plus Modified Pilates
*SPC*: Standard physiotherapy care
*SSI*: Symptom Severity Index
*UI*: Urinary incontinence

## References

[1] National Institute for Health and Care Excellence. The management of urinary incontinence in women. NICE clinical guidelines 40. Holborn London. 2013. https://www.nice.org.uk/guidance/cg171.

[2] Sinclair AJ, Ramsay IN. ﻿Review: the psychosocial impact of urinary incontinence in women.﻿ The Obstetrician & Gynaecologist. 2011;13:143–8. http://dx.doi.org/10.1576/toag.13.3.143.27665.

[3] National Institute for Health and Care Excellence. New NICE guidelines launched to improve treatment & care for millions of women suffering in silence. Holborn London. Ref: 2006/049; 2006. https://www.nice.org.uk/guidance/CG40.

[4] Hunskaar S, Vinsnes A (1991). The quality of life in women with urinary incontinence as measured by the sickness impact profile. J Am Geriatr Soc.

[5] Monz B, Pons ME, Humpel C, Hunskaar S, Quails D, Samsioe G (2005). Patient-reported impact of urinary incontinence - results from treatment seeking women in 14 European countries. Maturitas.

[6] Brown WJ, Miller YD (2001). Too wet to exercise? Leaking urine as a barrier to physical activity in women. J Sci Med Sport.

[7] Nygaard I, Girts T, Fultz NH, Kinchin K, Pohl G, Sternfeld B (2005). Is urinary incontinence a barrier to exercise in women?. Obstet Gynecol.

[8] Bø K, Sherburn M (2005). Evaluation of female pelvic-floor muscle function and strength. Phys Ther.

[9] Lee I, Shiroma EJ, Lobelo F, Puska P, Blair SN, Katzmarzyk PT (2012). Effect of physical inactivity on major non-communicable diseases worldwide: an analysis of burden of disease and life expectancy. Lancet.

[10] Patel A, Datta S, Kovoor ET. Pelvic floor muscle training versus other active treatments for urinary incontinence in women. Cochrane Database Syst Rev. 2008;(Issue 2):Art. No.:CD007173. 10.1002/14651858.CD007173.

[11] Culligan PJ, Scherer J, Dyer K, Priestley JL, Guingon-White G, Delvecchio D (2010). A randomized controlled trial comparing pelvic floor muscle training to a Pilates exercise program for improving pelvic muscle strength. Int Urogynecol J.

[12] Dumoulin C, Hay-Smith EC, MacHabée-Séguin G. Pelvic floor muscle training versus no treatment, or inactive control treatments for urinary incontinence in women. Cochrane Database Syst Rev 2014, Issue 5: Ar. No.:CD005654. DOI: 10.1002/14651858.CD005654.pub3, Issue 1. 10.1002/14651858.CD005654.pub3 24823491

[13] Savage AM (2005). Is lumbopelvic stability training (using Pilates model) an effective treatment strategy for women with stress urinary incontinence? A review of the literature and report of a pilot study. J Assoc Chartered Physiotherapists Womens Health.

[14] Chartered Society of Physiotherapy (2003). Quick reference guide: clinical guidelines for the physiotherapy management of females aged 16–65 with stress urinary incontinence.

[15] Demain S, Smith JF, Hiller L, Dziedzic K (2001). Comparison of group and individual physiotherapy for female urinary incontinence in primary care. Physiotherapy.

[16] Toozs-Hobson P, Loane K (2010). The psychology of incontinence: why successful treatments fail. J Assoc Chartered Physiotherapists Womens Health.

[17] Lee D, Lee LJ (2004). Stress urinary incontinence – a consequence of failed load transfer through the pelvis? Presented at the 5th world interdisciplinary congress on low back pain and pelvic pain.

[18] Kloubec J (2011). Pilates: how does it work and who needs it?. J Muscles Ligaments Tendons.

[19] Bø K, Herbert RD (2013). There is not yet strong evidence that exercise regimens other than pelvic floor muscle training can reduce stress urinary incontinence in women: a systematic review. J Phys.

[20] Anderson BD, Spector A (2000). Introduction to Pilates–based rehabilitation. Orthop Phys Ther Clin N Am.

[21] Black N, Griffiths J, Pope C (1996). Development of a symptom severity index and a symptom impact index for stress incontinence in women. Neurourol Urodyn.

[22] Patrick DL, Martin ML, Bushnell DM (2000). The I-QOL: a quality-of-life instrument specific to persons with urinary incontinence: User’s manual and scoring diskette.

[23] Kelleher CJ, Cardozo LD, Khullar V, Salvatore S (1997). A new questionnaire to assess the quality of life of urinary incontinent women. Br J Obstet Gynaecol.

[24] Avery K, Donovan J, Peters TJ, Shaw C, Gotoh M, Abrams P (2004). ICIQ: a brief and robust measure for evaluating the symptoms and impact of urinary incontinence. Neurourology and Urodynanmics.

[25] Rosenberg M (1965). Society and the adolescent self-image.

[26] Subak L, Whitcomb E, Shen H, Saxton J, Vittinghoff E, Brown JS (2005). Weight loss: a novel and effective treatment for urinary incontinence. J Urol.

[27] Danforth KN, Townsend MK, Lifford K, Curhan GC, Resnick NM, Grodstein F (2006). Risk factors forurinary incontinence among middle aged women. Am J Obstet Gynecol.

[28] Department of Health (2005). Mental capacity act.

[29] Hollander M, Wolfe D (1999). Nonparametric statistical methods.

[30] Lamb S, Pepper J, Lall R, Jørstad-Stein EC, Clark MD, Hill L (2009). Group treatments for sensitive health care problems: a randomised controlled trial of group versus individual physiotherapy sessions for female urinary incontinence. BMC Womens Health.

[31] R Core Team. R: A language and environment for statistical computing. Vienna: R Foundation for Statistical Computing. 2016. URL https://www.r-project.org; 2016.

[32] Ritchie J, Spencer L, Bryman A, Burgess RG (1994). Qualitative data analysis for applied policy research. Analyzing qualitative data.

[33] Ritchie J, Spencer L, O’Connor W, Ritchie J, Lewis J (2003). Carrying out qualitative analysis. Qualitative research practice: a guide for social science students and researchers.

[34] Lincoln YS, Guba EG (1985). Naturalistic inquiry.

