# Supplementary material for: Modified Pilates as an adjunct to standard physiotherapy care for urinary incontinence: a mixed methods pilot for a randomised controlled trial
Source: BMC Womens Health. 2018 Jan 12;18:16. doi: 10.1186/s12905-017-0503-y (PMC5767028; doi:10.1186/s12905-017-0503-y)
Supplement: Additional file 1: — Outcome measures - A description of the four outcome measures/questionnaires used: Symptom severity index (SSI)/Symptom Impact Index (SII); Incontinence quality of life questionnaire (I-QOL); International Consultation on Incontinence Questionnaire (ICIQ); Rosenberg self-esteem scale (RSE). (DOCX 25 kb) [file 12905_2017_503_MOESM1_ESM.docx]

***Symptom Severity Index*** (***SSI***) ***& Symptom Impact Index*** (***SII***) – were used to assess women’s views of the severity of their incontinence symptoms and the impact it has on their activities [23]. The items of both indexes had the same range of responses (from 0 to 4) so that each question contributed equally to the index score. For the *SSI*, the fourth question (i.e., *Number of activities that precipitate incontinence*) was made up of 9 dichotomous variables with 5 of them having a “not applicable” response available. Those patients who answered “yes” were given a score of 1, while a “no” or “not applicable” scored 0. The question was divided into five categories (0, 1-3, 4-5, 6-7, and 8-9) so that it could be added to the other 4 items in the SSI. For the *SII*, the fourth item, on the effects of symptoms on activities, was based on the proportion of applicable activities affected.

***Incontinence Quality of Life Questionnaire*** (***I-QOL***) – was used to evaluate the effects of incontinence symptoms on respondents’ life quality [24]. The survey consists of 22 questions with an additional section at the end designed to provide both general and incontinence-specific demographic information. There are three dimensions to the questionnaire: *Avoidance and Limiting Behaviour* (8 items, e.g., “I worry about not being able to get to the toilet on time”), *Psychosocial Impacts* (9 items, e.g., “I feel depressed because of my incontinence”), and *Social Embarrassment* (5 items, e.g., “I worry about others smelling urine on me”). Participants rated each statement on a 5-point Likert scale from 1 = *extremely* to 5 = *not at all*. The overall and subscale scores were computed by adding each item’s response, subtracting the lowest possible score and dividing that sum by the possible raw score range. The scores were then transformed to a have range from 0

=

*maximum problem* to 100

=

*no problem at all*. For the overall score, higher values indicate greater life quality, whereas for the subscales higher values represent less avoidant/limiting behaviours, psychosocial impact and social embarrassment.

**International Consultation on Incontinence Questionnaire** (***ICIQ***) – consisted of two questionnaires modules, namely *ICIQ – Urinary incontinence short form* (*ICIQ-UI sf.*) and *ICIQ – Lower Urinary Tract Symptoms Quality of Life* (*ICIQ-LUTSqol*).

***ICIQ-UI sf****.* – is a 4-item measure capturing the UI prevalence, severity, type and impact on quality of life [25]. The first two questions assess the frequency and volume of urine leakage, the third question the impact of UI on QoL, and the last one is an un-scored diagnostic item aimed to determine the type of UI. The value of the first three items is added together to produce a total severity score ranging from 0 to 21. Higher scores denote greater symptom severity.

***ICIQ-LUTSqol*** – assess the impact of urinary incontinence on quality of life with particular reference to social effects [26]. It is comprised of 20 questions divided into several domains: *role limitations* (2 items), *physical limitations* (2 items), *social limitations* (3 items), *personal relationships* (2 items), *emotions* (3 items), *Sleep/energy* (2 items), *severity measures* (4 items), *UI compromise* (1 item) and *general health perception* (1 item). Each question, excluding the last one, has the same range of responses (from 1 to 4). In addition to each question respondents are asked to rate the degree to which they were bothered by the impact of UI on a scale ranging from *0 = not at all* to *10 =* *a great deal*. The overall and subscale scores were computed by adding each item’s response, subtracting the number of items and dividing that sum by its highest possible value. The overall score range from 19 to 76, with higher values indicating increased impact on quality of life.

***Rosenberg Self-Esteem Scale*** (***RSE***) – measures state self-esteem by asking the respondents to reflect on their current feelings [27]. The scale consists of 10 statements, with 5 items measuring positive feelings (e.g., “I feel that I have a number of good qualities”) and 5 items assessing negative feelings about the self (e.g., “I feel I do not have much to be proud of”). Respondents are asked to indicate whether they agree with these statements or not on a 4-point Likert scale ranging from 0 = *strongly disagree* to 3 = *strongly agree* for the five positive phrased items and vice-versa for the negative ones. The value of each statement was added together to produce a total self-esteem score ranging from 0 to 30.
